# Association between socioeconomic status and academic performance in children and adolescents with chronic kidney disease

**DOI:** 10.1007/s00467-022-05515-3

**Published:** 2022-03-30

**Authors:** Rabia Khalid, Anita Van Zwieten, Siah Kim, Madeleine Didsbury, Anna Francis, Steven Mctaggart, Amanda Walker, Fiona E. Mackie, Chanel Prestidge, Armando Teixeira-Pinto, Belinda Barton, Jennifer Lorenzo, Suncica Lah, Kirsten Howard, Natasha Nassar, Eric Au, Allison Tong, Katrina Blazek, Jonathan C. Craig, Germaine Wong

**Affiliations:** 1 Centre for Kidney Research, The Children’s Hospital at Westmead, Sydney, Australia; 2 Sydney School of Public Health, The University of Sydney, Sydney, Australia; 3 Child & Adolescent Renal Service, Queensland Children’s Hospital, Brisbane, Australia; 4 Department of Nephrology, The Royal Children’s Hospital, Melbourne, Australia; 5 Department of Nephrology, Sydney Children’s Hospital at Randwick, Sydney, Australia; 6 Department of Nephrology, Starship Children’s Hospital, Auckland, New Zealand; 7 Children’s Hospital Education Research Institute, The Children’s Hospital at Westmead, Sydney, Australia; 8 Kids Neuroscience Centre, The Children’s Hospital at Westmead, Sydney, Australia; 9 School of Psychology, The University of Sydney, Sydney, Australia; 10 Menzies Centre for Health Policy and Economics, Faculty of Medicine and Health, University of Sydney, Sydney, Australia; 11 Child Population and Translational Health Research, Children’s Hospital at Westmead Clinical School, The University of Sydney, Sydney, Australia; 12 College of Medicine and Public Health, Flinders University, Adelaide, Australia; 13 Centre for Transplant and Renal Research, Westmead Hospital, Sydney, Australia

**Keywords:** Pediatric, CKD, Socioeconomic status, Education, Academic achievement

## Abstract

**Background:**

Lower socioeconomic status (SES) is associated with lower academic achievement; however, this relationship is understudied in children with chronic kidney disease (CKD). This study examined the relationship between SES and academic performance in children and adolescents with CKD.

**Methods:**

A total of 377 participants aged 6–18 years with CKD stages 1–5 (*n* = 199), on dialysis (*n* = 43) or with a kidney transplant (*n* = 135) were recruited. Five SES measures and a composite SES index were examined for associations with parent-rated average or above average academic performance in numeracy and literacy using multivariable logistic regression.

**Results:**

Participants’ median age was 12.6 years (IQR 8.9–15.5). Adjusted odds ratios (aOR) (95%CI) for better performance in numeracy and literacy, respectively, were 0.71 (0.44–1.15) and 0.75 (0.45–1.23) for children whose caregivers had lower educational attainment; 0.46 (0.26–0.80) and 0.53 (0.30–0.93) for lower household income; 0.52 (0.32–0.85) and 0.44 (0.26–0.73) for caregivers who were unemployed; 0.68 (0.41–1.12) and 0.59 (0.35–1.00) for caregivers with poor self-rated financial status; and 0.93 (0.53–1.64) and 1.00 (0.56–1.79) for caregivers who did not own their own home. Compared with the highest SES index quartile, the aORs for better performance by SES quartile in descending order were 1.24 (0.60–2.54), 0.76 (0.37–1.58), and 0.39 (0.18–0.86) for numeracy and 0.88 (0.41–1.85), 0.77 (0.35–1.66), and 0.32 (0.14–0.72) for literacy. No interactions were identified between SES and CKD stage, child age, or gender.

**Conclusions:**

Across all CKD stages, children from lower SES families are less likely to perform well in literacy and numeracy than those from higher SES households.

**Graphical abstract:**

A higher resolution version of the Graphical abstract is available as Supplementary information

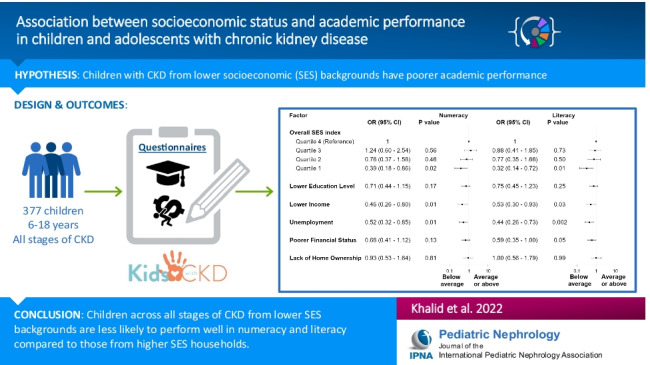

**Supplementary Information:**

The online version contains supplementary material including a graphical abstract available at 10.1007/s00467-022-05515-3.

## Introduction

Children and adolescents with chronic kidney disease (CKD) experience reduced survival, growth, and quality of life outcomes compared to children in the general population [1–4]. Children with CKD also experience cognition deficits in global cognition as well as the specific domains of executive function, attention, and memory [5]. Reduced academic performance is also observed in children with CKD, particularly for numeracy, reading, and spelling, with children treated with maintenance dialysis most affected [5, 6]. In addition to potential biological causes such as uremia, chronic anemia, hypertension, and reduced cerebral blood flow, other contributors may include missed school days and extra-curricular opportunities because of medical appointments, ongoing hospitalization, and long treatment duration with hemodialysis taking up to 60% of their school contact time [7–9]. Furthermore, the educational achievement gap observed in children with CKD may be further exacerbated by other social and economic factors.

Socioeconomic environments are associated with children’s academic achievement and educational attainment in the general population [10, 11]. This may be mediated by factors such as child health and nutrition, parenting styles, parental mental health, living conditions, and access to cognitively stimulating environments [12–14]. Academic achievement and educational attainment are important outcomes as they have critical impacts on later health and socioeconomic flourishing [10, 11]. In children with mild to moderate CKD, lower household income and maternal education have been associated with poorer academic achievement [15]. However, the association of family socio-economic status (SES) with academic performance has not been examined across the full spectrum of CKD. Understanding how socioeconomic factors influence educational outcomes in children with CKD is important as these children are already at an academic disadvantage, and factors that may compound this need to be identified to inform targeted interventions. We aimed to examine the association of socioeconomic disadvantage across multiple SES indicators with parent-rated educational outcomes in numeracy and literacy among children and adolescents with CKD, and to determine whether the association between SES and educational outcomes is modified by CKD stage.

## Methods

### Study design and population

This was a cross-sectional analysis of baseline data from the Kids with CKD (KCAD) study [16]. The KCAD study is a cohort study which takes a life-course approach to examine social and biological determinants of health and wellbeing among children and adolescents with CKD. The KCAD study design and methods have been detailed in the protocol and in previous publications [16–18]. From January 2012 to September 2016, families with a child aged 6–18 years with stages I–V CKD, on dialysis, or with a kidney transplant, were recruited through five of eight pediatric nephrology units in Australia and New Zealand. Participants were excluded if they were from families where no one spoke English, if informed consent could not be obtained from caregivers, or if the child was not undertaking formal education.

The Human Research Ethics Committee (HREC) approved this study at all participating centers (The Children’s Hospital at Westmead and Sydney Children’s Hospital (HREC/12/SCHN/159), Lady Cilento Children’s Hospital (HREC/12/QCRH/113), the Royal Children’s Hospital (Royal Children’s Hospital Human Research Ethics Committee: 33229) and Starship Children’s Hospital (New Zealand Health and Disability Ethics Committees: 15/NTB/37). Written informed consent (or assent depending on participant age) was obtained from all participants and/or caregivers.

### Exposures

A range of socioeconomic variables were collected as part of the key exposures of the KCAD study, through a self-reported questionnaire completed by the child’s caregiver. For this study, we assessed five measures of SES as exposures similar to our previous publication [16]: educational attainment (defined as primary school or secondary school or trade certificate versus other certificate/diploma or bachelor’s degree or higher education or other education (specification of “other” required in free text section)), gross household income (defined as below versus above the Australian median of $1,250 AUD per week or the New Zealand equivalent), employment status (defined as unemployed versus employed), home ownership (defined as owning a property with or without a mortgage: yes versus no), and perceived financial status, a subjective measure of financial deprivation [16, 19]. For perceived financial status, caregivers were questioned: “Given your current needs and financial responsibilities, would you say that you and your family are (1) Prosperous; (2) Very comfortable; (3) Reasonably comfortable; (4) Just getting along; (5) Poor; or (6) Very poor?”. This variable was dichotomized into (“just getting along”/ “poor”/ “very poor”) compared to (“reasonably comfortable”/ “very comfortable”/ “prosperous”). Consistent with our earlier publication [16], we also examined a global SES variable, a composite measure of the aforementioned SES factors. The global SES variable was generated using principal component analysis (PCA), a data reduction technique which compresses correlated variables into a smaller number of components with minimum loss of information. PCA was applied to all five individual SES variables (with > 10% contributions) to calculate a composite global SES index score [20]. Results of the PCA including the correlation matrix and component eigenvalues used to determine the global socioeconomic index scores can be found in a previous publication [16]. The global SES score was categorized into quartiles for analysis, with the highest quartile reflecting the highest SES.

### Outcome

Academic performance in numeracy and literacy was measured by parent-rated performance in Mathematics and English. Caregivers were asked: “Would you say your child’s performance in Mathematics is: well-below average, below average, average, above average?”. This question was repeated for the subject English. Parent-rated academic performance using a similar Likert scale has been shown to have moderate to large correlations with objective measures of academic performance (*r* is above 0.5) [21]. Other research exploring the precision of parental estimates of child test results also indicate moderate-high correlations with actual test results (*r* is above 0.5) [22–24].

### Covariates

Questionnaires were used to collect caregiver and child demographics including child age, sex, date of diagnosis with CKD, cause of CKD, CKD stage, learning difficulties, intellectual disability, ethnicity, postcode, private health insurance, and caregiver age, sex, marital status, and health. Medical information including cause and stage of CKD, medication, and comorbidities were collected through questionnaires and cross-checked with patients’ electronic medical records. Postcodes were used to classify the remoteness of participant locations using Australian Bureau of Statistics Remoteness Structure and the New Zealand Statistical Standard for Geographical Areas [25]. Australian major cities and New Zealand major urban areas were coded as “urban” with remaining cities being coded as “other” [26]. A validated subjective measure of overall health was used to measure caregiver health, with caregivers being asked: “In general, would you say your health is: poor, fair, good, very good, excellent?” [27–29]. Categorical variables with few cases were dichotomized into binary variables, including ethnicity (Caucasian versus non-Caucasian), child comorbidities (yes versus no), caregiver heath (poor/fair versus good/very good/excellent), and caregiver marital status (married/de-facto versus other).

### Analytical approach

All analyses were undertaken using R v3.6.3. Demographic characteristics were summarized as median (interquartile range (IQR)) for continuous variables and number (percentage) for categorical variables. To examine the association between SES and parent-rated academic performance, separate multivariable logistic regression models were fitted for each exposure: global SES index derived using PCA [16], education, income, employment status, home ownership, and perceived financial status. Due to small numbers for some categories, the 4-point Likert scale used to measure academic performance was collapsed to a binary variable: well-below average/below average versus average/above average (Supplementary Table 1). Covariate selection was based on an assumed causal model, informed by prior literature and expert knowledge and represented in a directed acyclic graph (DAG) (Supplementary Fig. 1). The variables CKD stage (coded as: stages I–V, dialysis, or transplant), child age, child gender, child ethnicity, caregiver age, caregiver marital status, and geographical location were included as forced covariates in all SES models on the basis of being known potential confounders. We also conducted sensitivity analyses adding in variables that may potentially act as mediators in addition to being potential confounders (comorbidities, cause of CKD, duration of CKD, private health insurance, and caregiver health). These variables were not included in the main models due to the risk of underestimating SES effects by controlling for potential mediators [30]. For the main models, we also assessed for interactions between the SES measure and the potential effect modifiers of CKD stage, child age, and child gender, using likelihood ratio tests. Where significant interactions were identified, we stratified the model by the effect modifier. For the association analysis, a *p*-value < 0.05 was considered statistically significant, and interaction analyses with *p*-value of *p* < 0.1 were explored. Given that there were 5 different SES measures, we applied Bonferroni corrections to account for multiple comparisons between CKD stage, gender, and age resulting in a new corrected $\alpha$-level of 0.006 for the interaction analyses. Observations with missing values were excluded from the models (percentage missing ranged between 13 and 17%).

## Results

### Participant characteristics and exposures

**Fig. 1:**
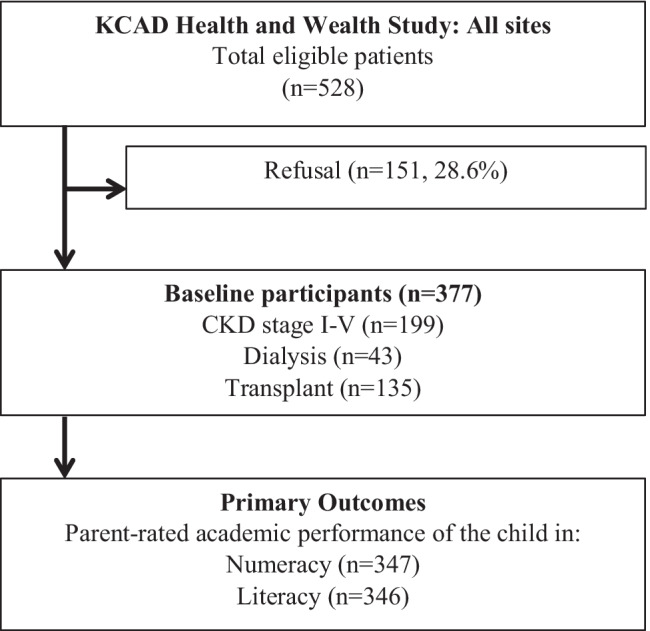
Recruitment and selection process

Of 528 eligible children and caregivers invited to take part in the study, 377 (71%) consented to participate (Fig. 1). Characteristics of the children and their caregivers are outlined in Table 1. The median age of the children was 12.6 years (IQR 8.9–15.5), 233 (62%) were male, and 220 (58%) were Caucasian. Most of the children had CKD stages I–V (*n* = 199, 53%), while 11% (*n* = 43) were on dialysis, and 36% (*n* = 135) had a kidney transplant. The average time since diagnosis with CKD was 8.2 years (SD: 5.1) and the most common cause of CKD was congenital anomalies of the kidney and urinary tract (*n* = 127, 34%). Almost two-thirds of the cohort had at least one comorbidity (*n* = 242, 64%) with the most common being hypertension (*n* = 120, 32%), followed by growth deficiency (*n* = 58, 21%), and behavioral issues (*n* = 58, 15%). Mild to severe caregiver-reported learning difficulties and intellectual disability were reported for 115 (30.5%) and 51 (13.5%) children and adolescents, respectively. The majority of the cohort resided in urban locations (*n* = 262, 70%), while only 36% (*n* = 135) had private health insurance. Among primary caregivers, the median age was 43.3 years (IQR 38.9–48.2) and 20% (*n* = 77) were single.

**Table 1:** Baseline characteristics of children with CKD and their caregivers (*N* = 377)

	***N* (%)**
Characteristics of children	
Gender	
Male	233 (61.8)
Age (years)	
6–9	120 (31.8)
10–14	142 (37.7)
15 +	115 (30.5)
Duration of CKD (years)	
Mean (SD)	8.2 (5.1)
Cause of CKD	
CAKUT	127 (33.7)
Glomerulonephritis	58 (15.4)
Nephrotic	92 (24.4)
Cystic	31 (8.2)
Other	69 (18.3)
CKD stage	
Stage I–V	199 (52.8)
Dialysis—peritoneal	18 (4.8)
Dialysis—hemodialysis	25 (6.6)
Transplant	135 (35.8)
Dialysis before transplant	104 (27.6)
Comorbidities	
Yes	242 (64.2)
Ethnicity	
Australian Indigenous	17 (4.5)
Māori	7 (1.9)
Pacific Islander	4 (1.1)
Caucasian	220 (58.4)
Middle Eastern	42 (11.1)
Asian	58 (15.4)
Other	22 (5.8)
Private health insurance	
Yes	135 (35.8)
State/country	
New South Wales	230 (61.0)
Victoria	67 (17.8)
Queensland	29 (7.7)
New Zealand	51 (13.5)
Geographic location	
Non-urban	113 (30.1)
Urban	262 (69.9)
Numeracy performance	
Below average	124 (32.9)
Average and above	223 (59.2)
Not reported	30 (8.0)
Literacy performance	
Below average	104 (27.6)
Average and above	242 (64.2)
Not reported	31 (8.2)
Learning difficulty	
None	260 (69.0)
Mild	60 (15.9)
Moderate	34 (9.0)
Severe	21 (5.6)
Intellectual disability	
None	322 (85.4)
Mild	25 (6.6)
Moderate	16 (4.2)
Severe	10 (2.7)
Characteristics of caregivers	
Age	
< 40 years	112 (29.7)
40–70 years	256 (67.9)
Gender	
Male	56 (14.9)
Marital status	
Single	77 (20.4)
Caregiver health	
Poor or fair	51 (13.6)
Good to excellent	324 (86.4)
Education	
Primary school/secondary school/trade certificate	160 (42.4)
Other certificate or diploma/other education/Bachelors degree or higher	215 (57.0)
Employment status	
Unemployed	156 (41.4)
Any employment	219 (58.1)
Perceived financial status	
Very poor to getting along	203 (53.8)
Comfortable to prosperous	171 (45.4)
Weekly household income	
< $1250 (AUD equivalent)	183 (48.5)
≥ $1250 (AUD equivalent)	173 (45.9)
Home ownership	
Rented/other	117 (31.0)
Owned outright/mortgage	255 (67.6)
Global SES index	
PCA quartile 1	286 (24.3)
PCA quartile 2	93 (26.3)
PCA quartile 3	90 (25.4)
PCA quartile 4	85 (24.0)

Caregiver characteristics presented are of the caregiver who completed the questionnaire. Percentages of total displayed include missing data: duration of CKD *n* = 1 (0.3%), dialysis before transplant *n* = 1 (0.3%), ethnicity *n* = 7 (2%), comorbidities *n* = 6 (2%), geographical location *n* = 2 (0.5%), learning difficulty *n* = 2 (0.5%), intellectual disability *n* = 4 (1%), caregiver age *n* = 9 (2%), caregiver gender *n* = 2 (0.5%), marital status *n* = 4 (1%), caregiver health *n* = 2 (0.5%), private health insurance *n* = 1 (0.3%), education *n* = 2 (0.5%), employment *n* = 2 (0.5%), financial status *n* = 3 (1%), income *n* = 21 (6%), home ownership *n* = 5 (1%), global SES index *n* = 23 (6%)

In terms of SES, 41% of primary caregivers (*n* = 156) were unemployed and 49% (*n* = 183) had weekly household income below $1,250. Over half had a non-trade certificate/diploma qualification, or bachelor’s degree or higher tertiary education, or other education (described by caregivers as “tertiary”) (*n* = 215, 57%), and around two-thirds owned a property either outright or with a mortgage (*n* = 255, 68%). For perceived financial status, 45% were comfortable to prosperous (*n* = 171). The majority of caregivers reported good to excellent health (*n* = 324, 86%). As expected, compared to caregivers of lower global SES, those in the highest SES quartile were more likely to be employed (*n* = 85, 100%), have higher education (*n* = 66, 77%), perceive themselves as comfortable to prosperous (*n* = 85, 100%), have private health insurance (*n* = 49, 58%), and have higher weekly household income (*n* = 85, 100%).

### Academic performance outcomes

Overall, parent-rated performance in numeracy and literacy was reported for 347 (92%) and 346 (92%) children, respectively. In the highest SES quartile, the proportion of parents rating their child’s performance as average or above was 59% for numeracy (*n* = 50) and 65% (*n* = 55) for literacy. The proportion of children with average or above average academic performance in the lowest SES quartile for numeracy was 50% (*n* = 43) and literacy was 55% (*n* = 47).

Table 2 displays academic performance across stages of CKD with additional stratification across stages I–II and III–V. The proportion of children with average or above average performance in numeracy and literacy, respectively, was 77% (*n* = 82) and 79% (*n* = 85) for those with CKD stages I–II; 58% (*n* = 53) and 58% (*n* = 53) for those with CKD stages III–V; 49% (*n* = 21) and 58% (*n* = 25) for those on dialysis; and 59% (*n* = 79) and 50% (*n* = 67) for children with a transplant.

**Table 2:** Academic performance of children across stages of CKD (*N* = 377)

		CKD stages I–II	CKD stages III–V	Dialysis	Transplant	Total
		*N* = 107	*N* = 92	*N* = 43	*N* = 135	*N* = 377
		*N* (%)	*N* (%)	*N* (%)	*N* (%)	*N* (%)
Academic performance						
Numeracy						
	Below average	17 (16)	32 (35)	17 (40)	45 (33)	111 (29)
	Average or above average	82 (77)	53 (58)	21 (49)	79 (59)	235 (62)
	Not reported	8 (7)	7 (8)	5 (12)	11 (8)	78 (21)
Literacy						
	Below average	13 (12)	33 (36)	13 (30)	58 (43)	117 (31)
	Average or above average	85 (79)	53 (58)	25 (58)	67 (50)	230 (61)
	Not reported	9 (8)	6 (7)	5 (12)	10 (7)	30 (8)

### Associations between family socioeconomic disadvantage and parent-rated academic performance

**Fig. 2:**
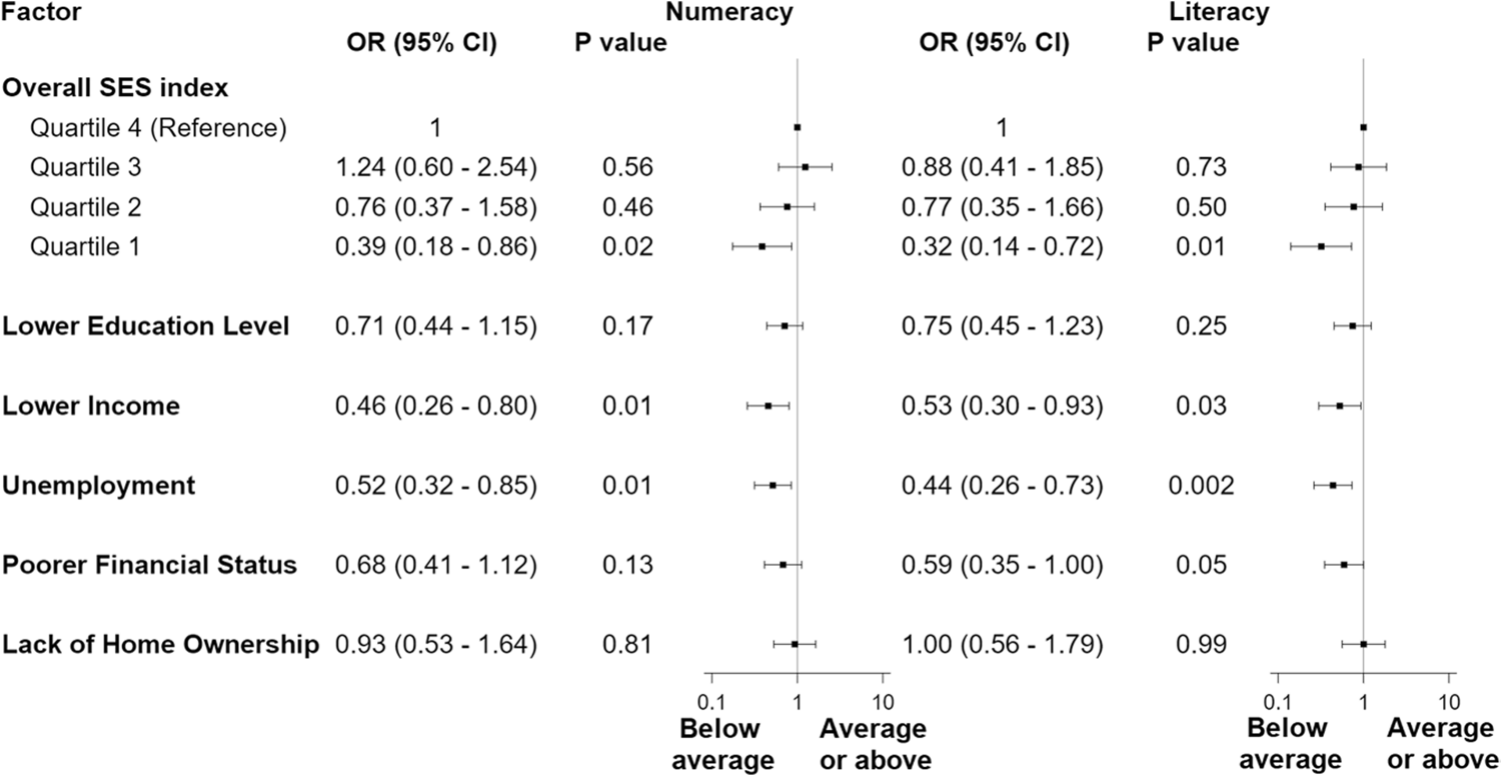
Associations between family socioeconomic disadvantage and parent-rated academic performance in numeracy and literacy. Each odds ratio is from a separate regression, adjusted for child age, CKD stage, ethnicity, gender, caregiver age, caregiver marital status, and geographic location. The overall SES index was derived from a principal component analysis on all 5 individual socioeconomic measures to form a composite global SES index, as described in “Methods.” It was a continuous variable that was categorized into quartiles for the regression models, with quartile 4 (highest SES) treated as the reference. In order from top to bottom, reference categories for the individual SES measures are: higher education level (other certificate or diploma/bachelor’s degree or higher/other education), higher income (> $1250 AUD/week), any employment, better financial status (comfortable to prosperous), and home ownership (owned outright/mortgage)

Figure 2 shows the association between SES measures and performance in numeracy and literacy adjusted for child age, CKD stage, ethnicity, gender, caregiver age, caregiver marital status, and geographic location. With reference to children from higher SES backgrounds, adjusted ORs (95%CI) for average or above average academic performance among children whose caregivers reported lower education level, lower household income, not being in paid employment, poorer financial status, and lack of home ownership were (i) 0.71 (0.44–1.15), 0.46 (0.26–0.80), 0.52 (0.32–0.85), 0.68 (0.41–1.12), and 0.93 (0.53–1.64), respectively, for numeracy, and (ii) 0.75 (0.45–1.23), 0.53 (0.30–0.93), 0.44 (0.26–0.73), 0.59 (0.35–1.00), and 1.00 (0.56–1.79), respectively, for literacy. With reference to the highest global SES quartile, adjusted ORs (95%CI) for average or above average performance by SES quartile in descending order were 1.24 (0.60–2.54), 0.76 (0.37–1.58), and 0.39 (0.18–0.86) for numeracy, and 0.88 (0.41–1.85), 0.77 (0.35–1.66), and 0.32 (0.14–0.72) for literacy.

### Interactions between SES and child CKD stage, age, and sex

There were no significant interactions between the six different SES measures and the variables of CKD stage, age group, or sex after accounting for multiple testing and comparisons.

### Sensitivity analyses

Results for the regression models were similar when adjusted for additional covariates that could act as potential mediators including comorbidities, cause of CKD, duration of CKD, private health insurance, and caregiver health (Table 3). With reference to children from higher SES backgrounds, adjusted ORs (95%CI) for better academic performance among children whose caregivers reported lower education level, lower household income, not being in paid employment, poorer financial status and lack of home ownership were as follows: 0.72 (0.42–1.21), 0.49 (0.27–0.89), 0.52 (0.30–0.89), 0.73 (0.42–1.27), and 0.85 (0.46–1.57) for numeracy, and 0.77 (0.45–1.31), 0.48 (0.26–0.89), 0.39 (0.22–0.68), 0.53 (0.30–0.95), and 0.85 (0.46–1.57) for literacy. With reference to the highest SES index quartile, adjusted ORs (95%CI) for better performance by SES quartile in descending order were 1.02 (0.48–2.17), 0.65 (0.30–1.41), and 0.37 (0.15–0.87) for numeracy, and 0.66 (0.30–1.46), 0.55 (0.24–1.24), and 0.25 (0.10–0.62) for literacy.

**Table 3:** Association between socioeconomic disadvantage and parent-rated performance in numeracy and literacy–sensitivity analysis adjusting for potential mediators and confounders

		Numeracy		Literacy		
Exposure	Odds ratio	(95% CI)	*p*-value	Odds ratio	(95% CI)	*p*-value
Overall SES index						
Quartile 4 (reference)	1	-	-	1	-	-
Quartile 3	1.02	(0.48–2.17)	0.964	0.66	(0.30–1.46)	0.310
Quartile 2	0.65	(0.30–1.41)	0.276	0.55	(0.24–1.24)	0.149
Quartile 1	0.37	(0.15–0.87)	0.024	0.25	(0.10–0.62)	0.003
Lower education level	0.72	(0.42–1.21)	0.212	0.77	(0.45–1.31)	0.334
Lower income	0.49	(0.27–0.89)	0.019	0.48	(0.26–0.89)	0.019
Unemployment	0.52	(0.30–0.89)	0.017	0.39	(0.22–0.68)	0.001
Poorer financial status	0.73	(0.42–1.27)	0.267	0.53	(0.30–0.95)	0.033
Lack of home ownership	0.85	(0.46–1.57)	0.607	0.85	(0.46–1.57)	0.607

Each odds ratio is from a separate regression, adjusted for child age, CKD stage, ethnicity, gender, caregiver age, caregiver marital status, geographic location and potential mediators and confounders: comorbidities, CKD cause, duration of CKD, private health insurance, and caregiver health. Global SES index was derived from a principal component analysis on all 5 individual socioeconomic measures, as described in “Methods.” It was a continuous variable that was categorized into quartiles for the regression models, with quartile 4 (highest SES) treated as the reference. In order from top to bottom, reference categories for the individual socioeconomic measures are: higher education level (other certificate or diploma/bachelor’s degree or higher/other education), higher income (> $1250 AUD/week), any employment, better financial status (comfortable to prosperous), and home ownership (owned outright/mortgage)

## Discussion

This study, of nearly 400 children representing all stages of CKD from the majority of pediatric nephrology centers in Australia and New Zealand, indicates that family SES is strongly and consistently associated with academic performance. Across the entire spectrum of CKD stages, children from the lowest quartile of the global SES index were around 60–70% less likely to perform well (average to above average) in numeracy and literacy compared to those from the highest SES quartile. CKD stage, age, and sex of the child did not appear to modify the relationship between SES and academic performance. Key individual drivers of poorer academic performance across SES domains included lower household income, lack of employment, lower self-perceived financial status, and lower education for the primary caregiver.

Research in the general population indicates that children from lower SES families are at risk of reduced academic achievement [14]. They are more likely to experience multiple stressors including poorer nutrition and health, and higher levels of stressful life events which can impact their brain development and key cognitive skills that underpin academic learning, such as problem-solving, working memory, planning, and attention [31–34]. However, the relationship between SES and academic performance has not been evaluated among children across the full spectrum of CKD. Previous research is limited to stages 3–5 CKD and suggests an association between income and maternal education and academic achievement [15]. Our research extends to children from all stages of CKD including those on kidney replacement therapy (KRT) and examines multiple measures of SES and confounders. We found that SES is associated with poorer achievement among children with CKD, which appeared to be driven largely by a threshold effect involving poorer performance for children in the lowest SES quartile. The lack of interactive effects by CKD stage suggested that socioeconomic disadvantage is equally detrimental to achievement for all children irrespective of the chronicity and severity of kidney disease. These findings are concerning given existing evidence that children with CKD may already have lower than average cognitive abilities and academic performance, with deficits in academic skills, executive function, and visual and verbal memory [5]. Our findings indicate that socioeconomic disadvantage may have additive, pervasive, detrimental effects on academic performance compared to having kidney disease alone.

As SES is a multidimensional construct, individual socioeconomic domains may vary in the strength of their association with academic achievement, and may influence academic outcomes via different mechanisms [12]. In our study, income and employment status showed the strongest associations with academic achievement in children with CKD. This association is consistent with previous research showing that income and employment are both considered markers of material resources [35]. Families with high income and employment may have more resources to support educational needs such as home schooling and tutoring, particularly for children with a chronic illness such as CKD where school absence is common [9, 36]. In contrast, no association was found for caregiver education and academic performance. Evidence from the general population has indicated that caregiver education is a strong predictor of academic performance [14, 37], which has been attributed to factors such as household literacy, caregiver teaching styles, and investment in educational resources [33]. The reasons for our finding are unclear, but may reflect the severity of the chronic illness and its long-lasting, persistent effects on life participation including schooling and education, irrespective of the education status and qualification of parents. Similarly, home ownership was not associated with academic performance in our cohort. Prior research has found that housing is a predictor for academic achievement, particularly for children from low SES backgrounds [37, 38]. Provision of a safe, affordable, stable, and quiet environment is a key strategy suggested for improving education outcomes among children living in poverty [39]. However, details and granularity of these constructs were not examined in our study, but should be a priority for future research.

Given the importance of childhood education for future SES, educational attainment, and health [10, 11], academic interventions should target children with CKD from low SES, with the goal of developing active, robust, and sustainable strategies to monitor progress and promote positive educational outcomes. Ongoing dialogue between caregivers, children, and clinicians, as well as teachers and education support staff is essential in setting attainable goals, particularly as priorities for outcomes can vary between these stakeholders [40]. Formal policies should be implemented to encourage communication in a proactive manner with a focus on prevention (rather than predominantly reactive or ad hoc practices) to ensure children are not precluded from learning opportunities due to factors such as school absences [41]. Strategies may include establishing appropriate electronic and remote leaning support, in addition to caregiver assistance with provision of resources and additional tutoring support. A previous review of academic interventions targeting children from low SES backgrounds found that tutoring, feedback and progress monitoring, and cooperative learning were effective, whereas other strategies including mentoring school personnel, increased resources, and incentive programs required further research [42]. The authors noted that they were unable to explain why some interventions were more effective than others, highlighting that the impact of an intervention will depend on local context. This reinforces that interventions would need to be modified to consider the impact CKD has both on children and their caregivers. To inform interventions, future research should further investigate the relationship between SES and academic performance in children with CKD with a focus on potential mediators. Aside from child health and the physical home environment, cognitive stimulation at home and parenting styles have been suggested to be key mediators between SES and child intellectual development [43].

This study had a number of strengths. Firstly, it was a large, multi-center study including children with varied SES backgrounds across all stages of CKD from 5 out of 8 pediatric nephrology units in Australia and New Zealand. SES is a multi-dimensional construct, and in the general population its associations with academic performance vary in strength across different SES domains [12]. As such, the use of numerous socioeconomic measures and the creation of a global composite index to examine the overall effects of SES across multiple domains is a major strength of this study [20]. We also included a number of potential confounders in our analyses, and built a separate model for variables that could act as mediators in order to avoid overadjustment bias. There are, however, some potential limitations. Given the observational and cross-sectional nature of the study, the impact of SES on academic performance over time could not be assessed, and there is a risk of residual confounding. The outcome measures, literacy and numeracy performance were measured subjectively via caregiver report, not actual test results, which may have introduced some measurement bias. However, similar measures have been shown to be associated with objective measures of academic performance [21]. For the SES exposures, education level and employment status were analyzed for only the primary caregiver, not for other caregivers, and household income was not adjusted for the number of household members, potentially underestimating their effects. There is a risk of selection bias as non-English speaking participants were excluded and 20% of eligible families refused to participate for unspecified reasons which may limit the generalisability of the results. Details on the granularity of comorbidities, premature births, and type and duration of dialysis before transplant were not collected and so the impact of these factors could not be examined. Finally, as the sample size of children on dialysis was small, our analysis may have been limited in its power to detect potential interactions with CKD stage.

Overall, these findings suggest that across illness severity, children with CKD from low SES backgrounds have poorer performance in numeracy and literacy than children who are more socioeconomically advantaged. Given the importance of educational outcomes for future health and socioeconomic attainment, there is a need for interventions targeted towards improving academic performance in children with CKD who are experiencing concurrent socioeconomic disadvantage. Further investigation into the relationship between SES and academic performance among children with CKD should systematically identify potential mediating factors to inform the development of academic interventions.

## Supplementary Information

Below is the link to the electronic supplementary material.

Graphical Abstract (PPTX 301 KB)

Supplementary file1 (DOCX 80 KB)
