# Supplementary material for: Association between socioeconomic status and academic performance in children and adolescents with chronic kidney disease
Source: Pediatr Nephrol. 2022 Mar 30;37(12):3195–204. doi: 10.1007/s00467-022-05515-3 (PMC9587100; doi:10.1007/s00467-022-05515-3)
Supplement: Supplementary file 1 — Graphical Abstract (PPTX 301 KB) [file 467_2022_5515_MOESM1_ESM.pptx]

## Slide 1
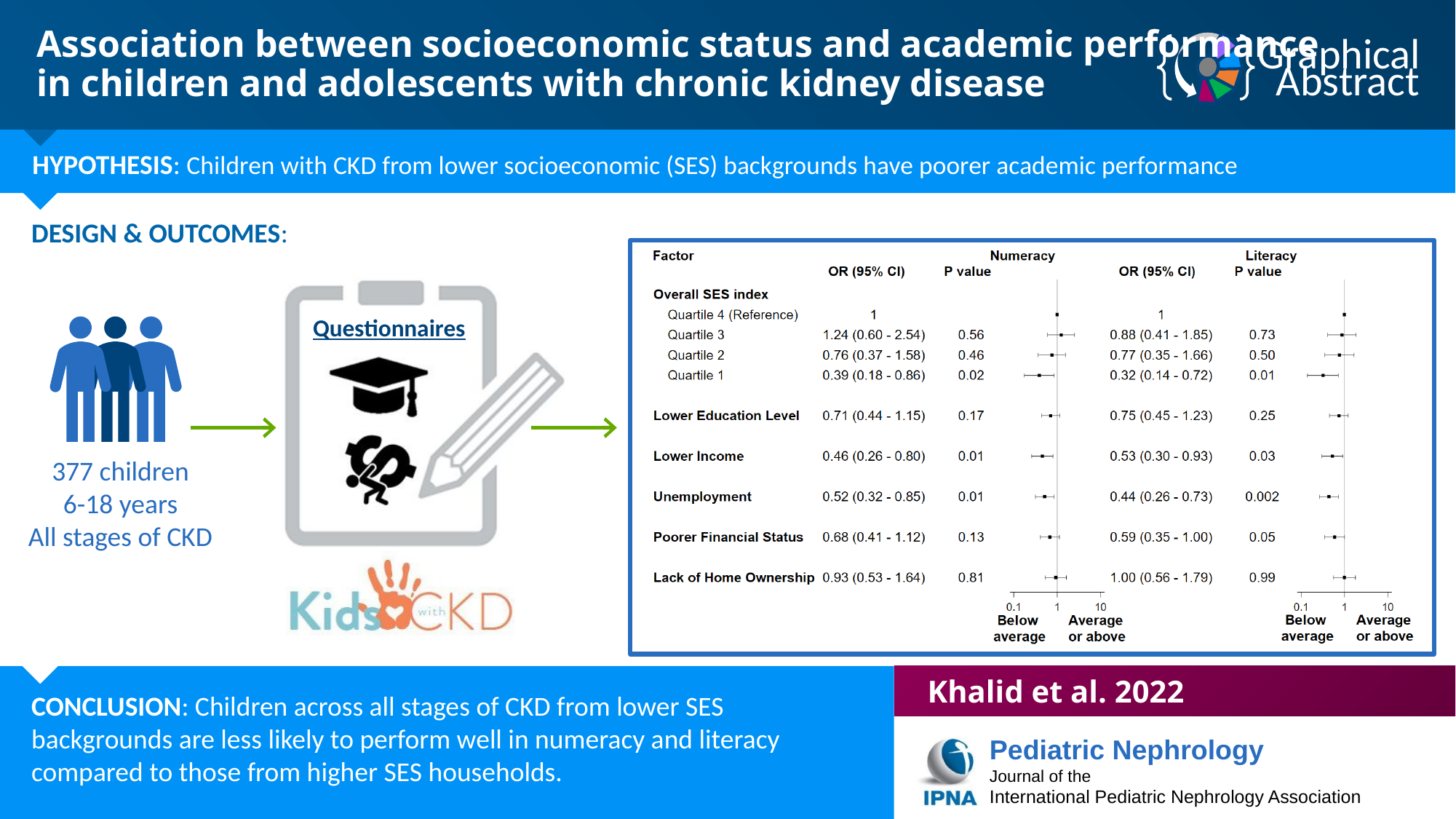

Association between socioeconomic status and academic performance
in children and adolescents with chronic kidney disease
HYPOTHESIS: Children with CKD from lower socioeconomic (SES) backgrounds have poorer academic performance
DESIGN & OUTCOMES:
Questionnaires
377 children
6-18 years
All stages of CKD
Khalid et al. 2022
CONCLUSION: Children across all stages of CKD from lower SES backgrounds are less likely to perform well in numeracy and literacy compared to those from higher SES households.
