# Supplementary material for: Association between socioeconomic status and academic performance in children and adolescents with chronic kidney disease
Source: Pediatr Nephrol. 2022 Mar 30;37(12):3195–204. doi: 10.1007/s00467-022-05515-3 (PMC9587100; doi:10.1007/s00467-022-05515-3)
Supplement: Supplementary file 2 — Supplementary file1 (DOCX 80 KB) [file 467_2022_5515_MOESM2_ESM.docx]

**Supplemental Figure 1 Directed acyclic graph**

**
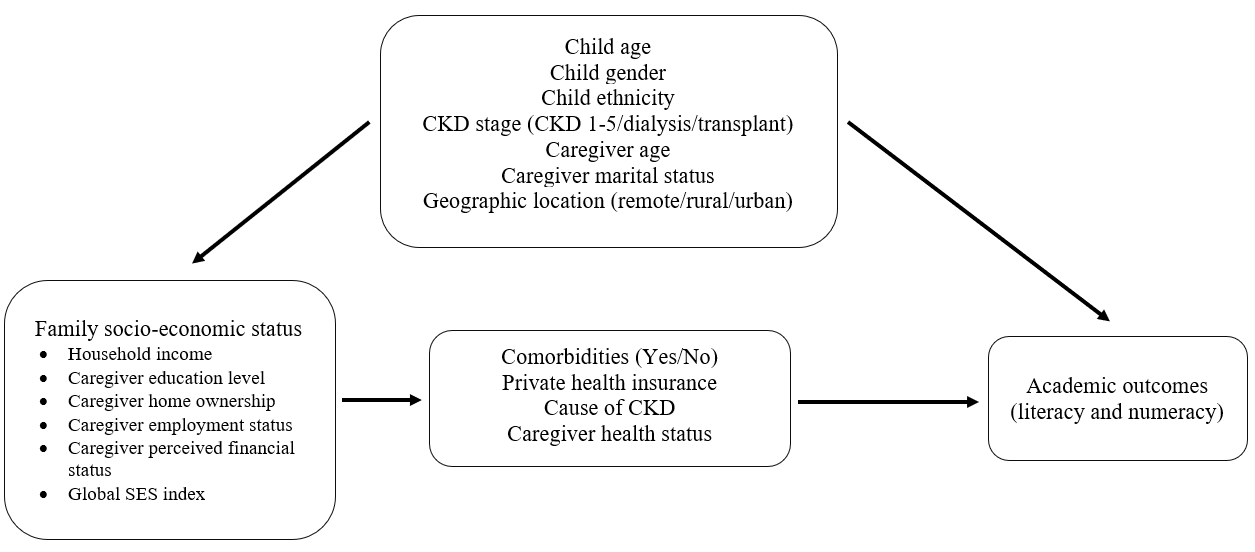
**

**Supplemental Table 1 Performance in numeracy and literacy across the global SES index**

|  |  | **Global SES quartile 1** | **Global SES quartile 2** | **Global SES quartile 3** | **Global SES quartile 4** | **Total** |
| --- | --- | --- | --- | --- | --- | --- |
|  |  | **N=86** | **N=93** | **N=90** | **N=85** | **N=377** |
|  |  | **N (%)** | **N (%)** | **N (%)** | **N (%)** | **N (%)** |
| **Academic performance** | | | | | | |
| **Numeracy** | | | | | | |
|  | Well below average | 15 (17) | 14 (15) | 9 (10) | 9 (11) | 47 (12) |
|  | Below average | 25 (29) | 17 (18) | 15 (17) | 14 (16) | 71 (19) |
|  | Average | 34 (40) | 38 (41) | 41 (46) | 27 (32) | 140 (37) |
|  | Above average | 9 (10) | 19 (20) | 19 (21) | 23 (27) | 70 (19) |
|  | Not reported | 3 (3) | 5 (5) | 6 (7) | 12 (14) | 26 (7) |
| **Literacy** | | | | | | |
|  | Well below average | 16 (19) | 14 (15) | 10 (11) | 6 (7) | 46 (12) |
|  | Below average | 22 (26) | 11 (12) | 12 (13) | 11 (13) | 56 (15) |
|  | Average | 42 (49) | 47 (51) | 41 (46) | 30 (35) | 160 (42) |
|  | Above average | 5 (6) | 16 (17) | 21 (23) | 25 (29) | 67 (18) |
|  | Not reported | 1 (5) | 5 (5) | 6 (7) | 13 (15) | 25 (7) |

Global SES index was derived using principal component analysis as described in the methods to create a composite measure based on 5 individual SES measures. It is a continuous variable categorized into quartiles with Quartile 4 being the highest SES group.
